# MRI as a biomarker for breast cancer diagnosis and prognosis

**DOI:** 10.1259/bjro.20220002

**Published:** 2022-05-26

**Authors:** Francesca Galati, Veronica Rizzo, Rubina Manuela Trimboli, Endi Kripa, Roberto Maroncelli, Federica Pediconi

**Affiliations:** ^1^ Department of Radiological, Oncological and Pathological Sciences, “Sapienza” - University of Rome, Viale Regina Elena, Rome, Italy; ^2^ Humanitas Clinical and Research Center – IRCCS, Via Manzoni 56 - 20089, Rozzano (MI), Italy

## Abstract

Breast cancer (BC) is the most frequently diagnosed female invasive cancer in Western countries and the leading cause of cancer-related death worldwide. Nowadays, tumor heterogeneity is a well-known characteristic of BC, since it includes several nosological entities characterized by diﬀerent morphologic features, clinical course and response to treatment. Thus, with the spread of molecular biology technologies and the growing knowledge of the biological processes underlying the development of BC, the importance of imaging biomarkers as non-invasive information about tissue hallmarks has progressively grown. To date, breast magnetic resonance imaging (MRI) is considered indispensable in breast imaging practice, with widely recognized indications such as BC screening in females at increased risk, locoregional staging and neoadjuvant therapy (NAT) monitoring. Moreover, breast MRI is increasingly used to assess not only the morphologic features of the pathological process but also to characterize individual phenotypes for targeted therapies, building on developments in genomics and molecular biology features. The aim of this review is to explore the role of breast multiparametric MRI in providing imaging biomarkers, leading to an improved differentiation of benign and malignant breast lesions and to a customized management of BC patients in monitoring and predicting response to treatment. Finally, we discuss how breast MRI biomarkers offer one of the most fertile ground for artificial intelligence (AI) applications. In the era of personalized medicine, with the development of omics-technologies, machine learning and big data, the role of imaging biomarkers is embracing new opportunities for BC diagnosis and treatment.

## Introduction

In recent years, with the spread of molecular biology technologies and the increasing knowledge about the biological processes underlying cancer development, considerable interest in biomarkers has progressively grown. In 2016, the latest glossary released by the U.S. Food and Drug Administration (FDA) - National Institutes of Health (NIH) Biomarker Working Group in its Biomarkers, Endpoints, and other Tools (BEST) Resource, defined a biomarker as “a defined characteristic that is measured as an indicator of normal biological processes, pathogenic processes or responses to an exposure or intervention, including therapeutic interventions. Molecular, histologic, radiographic or physiologic characteristics are types of biomarkers”.^1^ Moreover, the introduction of established tumor biomarkers in the most recent edition of Tumor Node Metastasis (TNM) staging system by the American Joint Committee on Cancer for several tumor entities, including BC, illustrates the movement in progress towards precision approaches and therapies.^2^ Within the framework of precision medicine, biomarkers become an important element for developing study methodology, research hypotheses and selectively applying scientific findings in cancer care.^1^ Imaging findings were only recently officially recognized as biomarkers even if it is in the intrinsic nature of imaging to be applied in this sense.^3^ BC was the most frequent cancer diagnosed among females in 2020 and breast MRI has been established as a non-invasive imaging modality for the detection, characterization and local staging of breast tumors with several recommendations including screening of high-risk females, pre-operative local staging and systemic therapy monitoring.^4–6^ Contrast-enhanced MRI (CE-MRI), diffusion-weighted imaging (DWI) and magnetic resonance spectroscopy (MRS)-based imaging biomarkers have shown to be highly correlated with BC molecular subtypes and other prognostic and predictive factors. Furthermore, multiparametric MRI (Mp-MRI) approaches have been introduced to investigate associations of imaging biomarkers with histological types and subtypes, response to treatment, risk of recurrence and overall survival in BC patients.^6–8^

## Biomarkers classification

**Figure 1.**
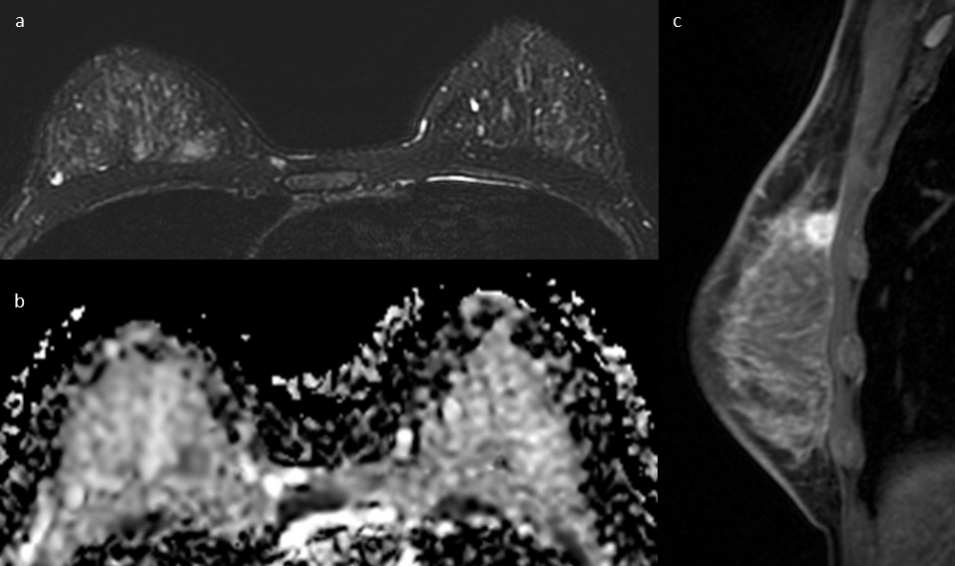
31-year-old female with triple-negative breast cancer of the right breast. (**a**) Axial fat-suppressed *T*_2_ weighted image shows a slight hyperintense round mass in the upper inner quadrant of the right breast, with mild intratumoral high signal intensity consistent with intralesional necrosis. (**b**) Axial ADC map shows a corresponding hypointense area of diffusion restriction. (**c**) Sagittal post-contrast *T*_1_ weighted image confirms the presence of a round mass lesion with rim enhancement. ADC, apparent diffusion coefficient.

Medical imaging can be a source of biomarkers in diagnostic, predictive, prognostic and monitoring settings.^1^ Breast imaging biomarkers can be divided into qualitative, ordinal and quantitative as shown in Table 1. Qualitative biomarkers are descriptive characteristics representative of the underlying pathologic condition.^1,9^ The American College of Radiology Breast Imaging-Reporting and Data System (ACR BI-RADS) lexicon is the first and the best validated system of imaging descriptors in radiology.^10^ Ordinal biomarkers are categories with intrinsic rankings that can be arranged in a meaningful order.^9^ Breast MRI background parenchymal enhancement (BPE) with minimal, mild, moderate and marked categories is an example of ordinal biomarkers. Quantitative biomarkers are objective, measurable and reproducible parameters.^9^ Anatomic structures 2D and 3D measurements are examples of quantitative biomarkers essential in diagnosis, staging and monitoring of response to treatment, as it is when applying the Response Evaluation Criteria in Solid Tumors (RECIST 1.1) criteria.^11^ Additional quantitative biomarkers derived from breast Mp-MRI, are apparent diffusion coefficient (ADC) maps values of DWI and the transfer constant (K_trans_) that provides a measure of capillary permeability with CE-MRI perfusion. The use of panels or scoring systems combining multiple imaging parameters, such as TNM, can perform significantly better than individual ones.^12,13^ Further example is the increased diagnostic performance of Mp-MRI in BC molecular subtype prediction based on the underlying biological features. For instance, well-known imaging biomarkers of triple-negative BC (TNBC) are intralesional necrosis and peritumoral edema at *T*_2_ weighted images, smooth margin and rim enhancement at CE-MRI^14–17^ Figure 1. In recent years, researches have demonstrated that different BC phenotypes show specific imaging texture features.^18^ Thus, the new perspective of breast MRI includes artificial intelligence (AI) applications. The intrinsic multiparametric nature of MRI has the greatest potential to incorporate AI applications into the so called precision medicine. The number of breast imaging biomarkers will increase in the next future, expanding the role of imaging in breast care.

**Table 1.** Brief description of the different types of biomarkers and respective examples

Category of biomarkers	Characteristics	Examples
Qualitative	Descriptive characteristics that are visually assessed	BI-RADS descriptors (*e.g.* tumor shape and margins, mass or non-mass lesion, enhancement morphology)
Ordinal	Descriptive characteristics that can be arranged in ranks	BPE
Quantitative	Characteristics susceptible of quantitative assessment with a unit of measurement	RECIST criteria (linear and volume measurement), ADC value, K_trans_, K_ep_, DTI, IVIM, DKI (D value, K value), tCho, PE/PC ratio

ADC, apparent diffusion coefficient; BPE, background parenchymal enhancement; DKI, diffusion-weighted kurtosis; DTI, diffusion tensor imaging; IVIM, intravoxel incoherent motion; PC, phosphocholine; PC, phosphoethanolamine.

## Techniques

Table 2 shows different biomarkers classified according to imaging modality.

**Table 2.** Different biomarkers classified according to imaging modality

Imaging modality		Biomarkers
CE-MRI		Morphologic features
		Enhancement T/I kinetics
		BPE
		Transfer constant from the vascular space to the tumor interstitium (K_trans_)
		Transfer constant from the interstitium to the blood plasma (K_ep_)
		Extravascular–extracellular volume fractions (Ve)
DWI		ADC value
	IVIM	Tissue diffusivity, tissue microcapillary perfusion
	DTI	Directional diffusivity of water molecules
	DKI	D value, K value
MRS		Total choline peak (tCho)
		PE/PC ratio
		Lipid concentration

ADC, apparent diffusion coefficient; BPE, background parenchymal enhancement ; DKI, diffusion-weighted kurtosis; DTI, diffusion tensor imaging; IVIM, intravoxel incoherent motion; PC, phosphocholine; PE, phosphoethanolamine; TI curve, time/intensity curve.

### CE-MRI

Over the past two decades, CE-MRI has improved breast MRI diagnostic accuracy with sensitivity up to 99% and variable specificities ranging from 47 to 97% in the detection and characterization of breast lesions,^9,19^ assessing breast tissue vascular microenvironment and tissue permeability. As angiogenesis plays an important role in tumor biology, CE-MRI biomarkers and pharmacokinetic parameters were widely investigated.^20^ BPE is described as the enhancement of fibroglandular tissue in the dynamic CE-MRI early phases.^21,22^ In the last decade, BPE has generated interest and has been added in the last edition of BI-RADS MRI lexicon that provides standard descriptors for BPE level and distribution.^10,23^ It has been shown that BPE is a hormonally sensitive feature that declines over time with the onset of menopause, after oophorectomy and in patients who have been treated with tamoxifen or aromatase inhibitors.^24,25^ Initial results from two case–control studies on high-risk subjects have attested that BPE can be a predictive biomarker of BC risk. In particular, in females previously stated as at high risk of BC, a marked BPE increases the personal risk of BC up to tenfold.^21,26^ Kim et al found a significant correlation between qualitative BPE and epidermal growth factor receptor (EGFR)-positive BCs compared to EGFR-negative BCs. In this paper, BPE was also measured with the semi-quantitative background enhancement coefficient (BEC), evaluated through regions of interest drawn on healthy breast tissue. BEC as well as ipsilateral whole breast vascularity, were significantly higher in >2 cm tumors than in tumors smaller in size.^27^ In addition, BPE may reduce breast MRI sensitivity by obscuring enhancing cancers or may decrease specificity by determining enhancement patterns that mimic the appearance of malignancies.^28,29^ Nevertheless, current evidences have not confirmed a significant correlation between BPE and an increase in either false-positive or false-negative findings on breast MRI.^22,30^ However, a recent systematic review highlights the wide variability in the quantitative evaluation of BPE on breast MRI, thus uniform criteria should be defined to consolidate BPE as a biomarker.^31^

Furthermore, in the era of new cellular signaling pathways and molecular therapies, CE-MRI can be used for quantitative assessment of the vascular microenvironment and the tissue permeability.^9^ Breast lesions kinetic patterns differ between malignant and benign lesions; thus, enhancement time/intensity curve characteristics can be used in combination with morphologic features to improve differential diagnosis. Semi-quantitative parameters can be extracted from the enhancement curves, including the onset time, maximum signal intensity, gradient or rate of contrast uptake and washout, and initial area under the time signal curve.^32,33^ In recent years, the associations between contrast-enhancement kinetics and molecular subtypes were widely investigated.^32,34^ According to Blaschke and Abe,^32^ HER2 positive tumors demonstrated a faster and earlier enhancement than other subtypes, while luminal A and basal cancers showed a reduced washout during the delayed phase. This can be attributed to the frequent association of luminal A cancers with ductal carcinoma *in situ*, which rarely demonstrates washout kinetics; while basal subtype cancers are often characterized by tumoral necrosis and central scarring, which typically shows a persistent enhancement.^32^ Quantitative analysis involves pharmacokinetic modeling and requires more complex methods for estimating changes in tissue contrast agent concentration following intravenous injection. The transfer constant, K_trans_, describes the transendothelial transport of contrast medium by diffusion from the vascular space to the tumor interstitium and provides a measure of vascular permeability. Gradually, gadolinium diffuses back into the vascular system, with K_ep_ representing the transfer constant from the interstitium to blood plasma and Ve the extravascular–extracellular volume fraction. K_trans_ and K_ep_ are generally high in tumors. A significant reduction up to a third has been detected in both parameters in patients with locally advanced BC early responding to NAT,^35^ while an increase in Ve has been shown in non-responders.^36^ According to the authors O’Flynn and Nandita M. de Souza,^33^ K_trans_ can be used as a predictive biomarker to evaluate response to antiangiogenic drugs or vascular disruptive agents such as bevacizumab, a humanized monoclonal antibody directed against the vascular endothelial growth factor (VEGF), with a change in K_trans_ value >40% commonly considered as the threshold for definitive disease response.^37^ In summary, the available literature shows an ample consensus on the diagnostic value of CE-MRI measurements for non-invasive characterization and prognostication of BCs as well as for therapy monitoring during NAT.

### DWI

**Figure 2.**
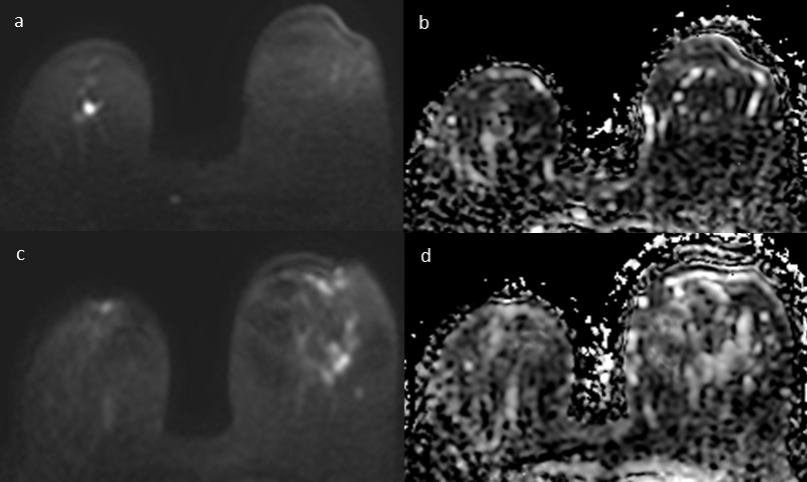
54-year-old female with bilateral breast cancer, invasive ductal carcinoma on the right breast and ductal carcinoma *in situ* on the left breast. (**a**) Axial DWI image (b value = 1000 s/ mm^2^) shows an hyperintense lesion between the upper quadrants of the right breast, (**b**) with corresponding 0,8 × 10^−3^ mm^2^/s ADC values. (**c**) Axial DWI image (b value = 1000 s/mm^2^) shows an hyperintense area in the upper outer quadrant of the left breast, (**d**) with higher ADC values (1,02 × 10^−3^ mm^2^/s). ADC, apparent diffusion coefficient; DWI, diffusion-weighted imaging.

**Figure 3.**
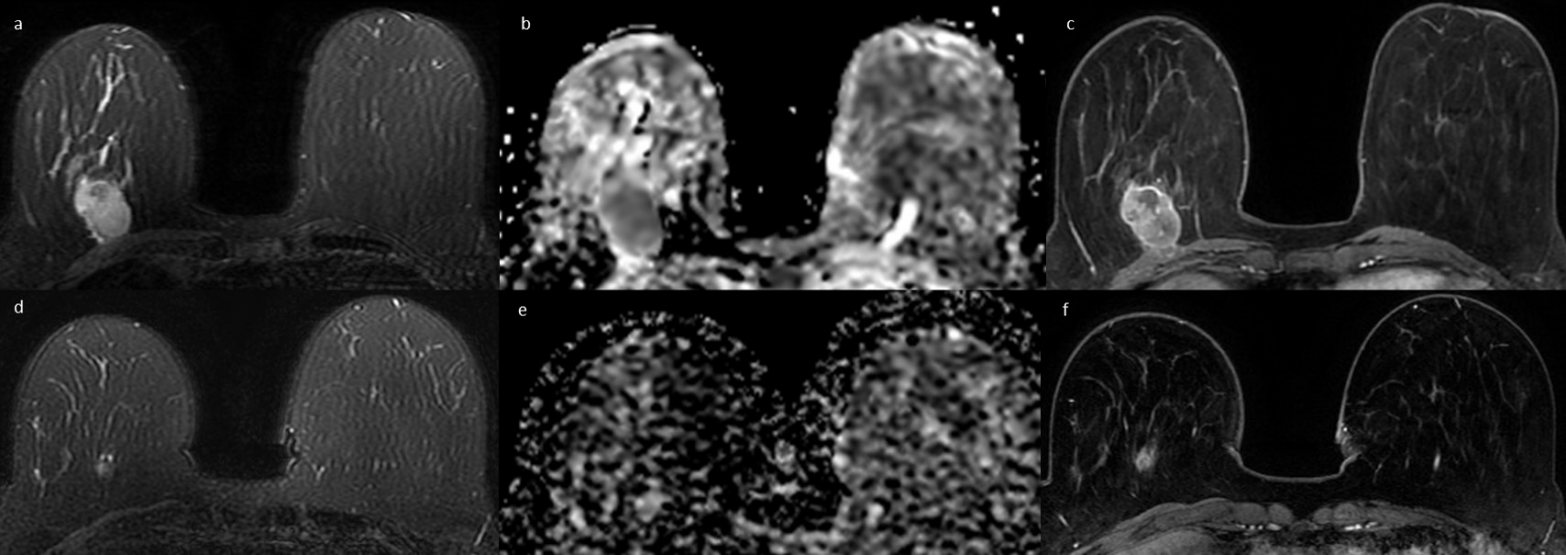
61-year-old female with triple-negative breast cancer of the right breast, before (**a,b,c**) and after 3 months (**d,e,f**) of neoadjuvant chemotherapy. (**a**) Axial fat-suppressed *T*_2_ weighted image shows a 27 mm hyperintense oval mass with regular margins between the lower quadrants of the right breast. (**b**) The mass appears homogeneously hypointense in the ADC map with 0,7 × 10^−3^ mm^2^/s ADC value. (**c**) Axial post-contrast *T*_1_ weighted image shows a corresponding oval mass with rim enhancement. (**d**) Axial fat-suppressed *T*_2_ weighted image shows a reduction in size of the lesion, which appears as a round hyperintense mass with blurred margins. (**e**) The ADC map shows a hypointense lesion with increased ADC values (1,2 × 10^−3^ mm^2^/s). (**f**) Axial post-contrast *T*_1_ weighted image shows a residual 12 mm round mass lesion. ADC, apparent diffusion coefficient.

A review of the literature emphasizes DWI as a potential source of biomarkers to increase breast MRI specificity, significantly improving diagnostic accuracy and reducing unnecessary biopsies.^38^ DWI explores different functional tissue features including water molecules motion in the extracellular space, density of neoplastic cells, tissue microstructure, cell wall integrity and permeability. Compared to normal gland, tumor tissue is characterized by a lower water molecules diffusion and, consequently, lower ADC values due to the high cell density and the presence of numerous intra- and intercellular membranes, thus the ADC map allows to quantitatively evaluate diffusivity of water molecules.^4^ In recent years, the association between ADC value and standard histopathological and immunohistochemical breast tumor features such as histological type, grade, hormonal receptor and Ki-67 expression, HER2 status, were widely investigated.^39–42^ Bickel et al tested DWI as an imaging biomarker to differentiate ductal carcinoma *in situ* from invasive lesions.^43^ Authors demonstrated that ADC value ≤ 1.01 10^−3^ mm^2^/s allows the identification of invasive tumors with 78% sensitivity and 90% specificity (Figure 2). In the same research, no significant differences in ADC values were found between high- and low-grade tumors, in contrast to a previous study in which a correlation between high histopathological grade (G3) and low median ADC values was found.^39^ Subsequently, a significant association was found between high ADC values and luminal A subtype.^40^ Guvenc et al described a correlation between low ADC values and more aggressive subtypes of BC, secondary to high cell density.^42^ In particular, a statistically significant relationship was found between low ADC values and low hormone receptors positivity along with the presence of abnormal lymphnodes. In 2007, Hamstra et al^44^ first introduced DWI as a biomarker to assess the response to NAT in different types of cancer, including BC. Significant preclinical and clinical studies were performed to support the hypothesis that DWI was a promising biomarker for early evaluation of response to NAT. ADC values variations may give early information regarding response to therapy, due to ADC peculiarity to reflect tumor cellularity and necrosis status.^45,46^ Park et al^47^ found an association between BCs pre-treatment low ADC value and better response to chemotherapy. The accuracy of ADC in predicting the response to NAT was evaluated by Richards et al^48^ who concluded that pre-treatment tumor ADC values varied according to breast tumor phenotypes and were predictive of pathologic response in TNBCs (Figure 3).^49–51^ However, the wide variability of results in the literature and the lack of standardization are two major limitations of DWI and DWI-derived biomarkers. To overcome these drawbacks, the European Society of Breast Imaging (EUSOBI) has established a multicenter, international working group composed of clinical experts, MRI physicists and MRI equipment suppliers with experience in breast DWI.^52^ DWI working group objectives include the promotion of DWI in MRI protocols, the diffusion of technical guidance for DWI protocols and the creation and improvement of quality control methods, to finally find agreement on the optimal image processing, visualization and interpretation. In a recent review, Iima et al addressed advanced DWI models, such as intravoxel incoherent motion (IVIM), diffusion tensor imaging (DTI) and diffusion weighted kurtosis (DKI).^38^ IVIM is a biexponential model that simultaneously evaluates tissue diffusivity and tissue microcapillary perfusion. DTI gives quantitative data on the water molecules directional diffusivity in biological tissues. The information obtained about diffusion anisotropy could be a potential biomarker of malignancy. It has been hypothesized that proliferating neoplastic cells, which generally destroy the normal structure of the mammary gland, could reduce anisotropy. DKI quantifies the incoherent movement of water molecules and tissue microperfusion typical of non-Gaussian phenomena, useful in the detection and characterization of breast lesions. A model proposed to quantify the Gaussian and non-Gaussian diffusion is able to estimate the D value, which represents the Gaussian diffusion, and the K value, a kurtosis parameter that represents the deviation from the Gaussian diffusion. These technological advances are supported by several studies and revealed to be useful in establish benign or malignant nature of breast lesions, in evaluating Ki-67 and tumor grading and in predicting treatment response. DWI is a promising qualitative and quantitative biomarker, a valid tool in lesion characterization and therapy monitoring. However, standardization of the acquisition and interpretation modalities of the extracted DWI data will enhance its clinical value.

### MRS

MRS is a non-invasive functional technique that provides information on biochemical changes in specific anatomic structures by identifying and monitoring the chemical composition of the tissue under examination. In the last decades, proton MR spectroscopy (^1^H-MRS) based on the detection of the total choline peak (tCho) has been implemented in breast Mp-MRI protocols, since several studies reported higher levels of tCho in BCs compared to benign lesions and normal breast tissue.^53,54^ Therefore, on the basis of different choline peaks, MRS is a potential biomarker to improve diagnostic accuracy and predict tumor aggressiveness.^55^ The diagnostic accuracy of a high-spatial-resolution 3D ^1^H-MRS protocol at 3 T was evaluated for the characterization of breast lesions, based on tCho signal-to-noise ratio threshold levels and proved its potential in becoming a valuable adjunct to CE-MRI in distinguishing between benign and malignant breast lesions.^56^ Other authors demonstrated that tumor tCho measurements were significantly higher in invasive ductal carcinomas vs *in situ* cancers and that tCho correlated with numerous prognostic factors, including histologic and nuclear grade, and estrogen receptor status.^57^ Thus, the addiction of MRS in multiparametric protocol leads to significantly higher diagnostic accuracy than CE-MRI, also significantly reducing false-negative and false-positive cases.^58^ However, tCho sensitivity significantly decreases for smaller cancers, due to insufficient detection of tCho signal.^59^ Available data suggest tCho as a potential biomarker for treatment response assessment and early prediction of the final NAT effect. In treated lesions, an early decrease in tCho levels, after the initial course of therapy, is consistent with tumor response and is even more sensitive than other morphological and functional criteria. Instead, it was demonstrated an increase in tCho concentration in patients with local recurrence.^60–62^ Beyond tCho further metabolites can be detected and monitored, above all, the most promising seems to be the assessment of lipid metabolism. Thakur et al demonstrated that intratumoral lipid concentration allows to distinguish benign from malignant tumors and to differentiate among BC molecular subtype.^63^ Ramadan and colleagues^64^ described that healthy breast tissue in patients with BRCA-1 and BRCA-2 mutation was likely to differ from non-mutation carriers in unsaturated fatty acids triglycerides and cholesterol levels. Further studies are needed to support these results, that could open new scenarios in high-risk females screening programs. Recently, phosphorus spectroscopy (^31^P MRS) has been introduced as a new functional MRI parameter for BC diagnosis and therapy monitoring. In particular, it has been demonstrated that a decrease in the phosphoethanolamine/phosphocholine (PE/PC) ratio is a sensitive cellular-level indicator of malignancy.^65^ Moreover, changes in PE/PC ratios are observed during NAT. These promising results of ^31^P MRS have been obtained with 7 T-scanners. Thus, due to the lack of data collected with ultra-high field scanners certainly related to their limited diffusion, the use of ^31^P MRS as a breast MRI biomarker is still limited in clinical practice.

## Artificial intelligence

AI is a computer science branch able to analyze a multitude of complex data. In recent years, AI potential has been exploited in diagnosis, treatment and outcome prediction of many clinical conditions, including BC. Breast MRI, due its intrinsic multiparametric concept, is inherently suitable for AI applications. Each breast MRI generates multiple volumes of images that can be integrated and arranged according to the different diagnostic, therapeutic or prognostic purposes.^66^ CE-, *T*_1_- and *T*_2_ weighted, DWI and MRS images provide large datasets fitting AI applications and potential MRI biomarkers. In Gilles et al landmark paper was clearly stated that “images are more than pictures, they are data”, focusing on the hidden power of imaging, including information not always perceptible by human eye.^67,68^ The term “radiomics” was first used in 2010, to describe the process of building predictive models via quantitative data extracted from radiological examinations. Radiomics consists of different stages, which includes image acquisition, reconstruction, segmentation and rendering, features extraction and qualification, database and data sharing for any *ad hoc* computer analysis.^69^ The goal of radiomics is to provide valuable diagnostic, prognostic or predictive information derived from biological and medical data.^70^

In a review published in 2018, radiomics models based on different imaging methods including MRI were investigated. Studies that analyzed BC using a radiomic approach and that provided data on BC diagnosis (detection or characterization), BC prognosis (response to therapy, morbidity, mortality) or provided data on technical challenges (software application: open source, repeatability results) were included. Authors concluded that the application of radiomics in BC patients was an emerging translational research topic, with the capability of improving the knowledge of the breast lesions specifics.^71^ Currently, radiomics encounters many obstacles: the need for large clinical data and standardized protocols, the dispersion of data in different centers, the excessive costs for technological development. In recent years, several countries have already adopted many approaches to control variability in clinical trial protocols, data acquisition and analysis. For instance, across Europe, consistent protocol guidance was achieved with the help of European Association of Nuclear Medicine. The Quantitative Imaging Biomarker Alliance initiative also aims to reach the same task in a much broader level. The known clinically significant genetic variables in BC and the good time and spatial resolution of breast MRI provide an excellent basis for radiogenomics research.^72^ At present, breast imaging radiogenomics has primarily centered on CE-MRI sequences, focusing on differentiation of molecular subtypes and assessment of recurrences.^73^ Several authors identified an association between different radiomic tumor phenotypes and various genomic features involved in multiple layers of molecular regulation and gene expression profiles of BCs.^74,75^ Other authors^76^ investigated possible correlation between imaging features and prognostic genomic tests such as Oncotype Dx, concluding that breast MRI has the potential to serve as a source of imaging biomarkers in the prediction of cancer recurrence. Further studies demonstrated a correlation between the expression of some genetic markers and the MRI variables during NAT although these results are still preliminary. A recent literature review^77^ found that radiogenomics, combining genomic information with emerging deep learning (DL) modalities, could predict the effectiveness of NAT and provide information on disease progression. Among AI applications specific methodologies are machine learning (ML) and DL.^66^ ML is a technology that allows the automatic training of machines with the aim of obtaining predictive data set based on the data and algorithms provided, without explicit programming. DL, a subset of ML, is characterized by a high accuracy, less need of human intervention but requires a huge amount of training data and expensive hardware and software.^78^ Due to MRI intrinsic multiparametric nature, ML application in breast MRI is fast-paced developing and many studies are demonstrating ML usefulness in lesion detection and classification, prediction of NAT response and recurrence risk, and therefore to guide therapeutic decisions.^79,80^ DL demonstrated high diagnostic accuracy to differentiate benign from malignant lesions,^79^ improving the diagnostic performance of breast MRI by decreasing the false positives and improving the positive-predictive value.^81^ Furthermore, DL has also been exploited extensively for evaluating the response to NAT.^77^

The integration of AI into breast imaging may enable the creation of new imaging biomarkers that incorporate patient clinical and tumor structural characteristics. Moreover, biomarkers could be incorporated into patient risk stratification via personalized imaging.^82^ Nevertheless, AI applications are not ready to be incorporated into clinical practice, nor to replace expert human observers with the ability to critically evaluate MRI images and patient history.

## Conclusions

Breast MRI may act as a diagnostic and prognostic tool to improve BC management through the extraction of a plenty of functional cancer parameters serving as imaging biomarkers. The intrinsic multiparametric nature of MRI provides specific information to visualize and quantify the functional processes of cancer development and progression, in order to improve detection and characterization of breast lesions, monitoring and prediction of response to therapy, and differentiation of biological BC subtypes. Moreover MRI images, due to their complex information content, are a fertile ground for AI applications. These may improve the integration of imaging biomarkers in clinical decision-making through the building of accessible predictive integrated models aiming at individualized medicine.

## References

[1] Weaver O, Leung JWT . Biomarkers and imaging of breast cancer. AJR Am J Roentgenol 2018; 210: 271–78. doi: 10.2214/AJR.17.18708 29166151

[2] Bolan PJ, Kim E, Herman BA, Newstead GM, Rosen MA, Schnall MD, et al . MR spectroscopy of breast cancer for assessing early treatment response: results from the ACRIN 6657 MRS trial. J Magn Reson Imaging 2017; 46: 290–302. doi: 10.1002/jmri.25560 27981651 PMC5464996

[3] O’Connor JPB, Aboagye EO, Adams JE, Aerts HJWL, Barrington SF, Beer AJ, et al . Imaging biomarker roadmap for cancer studies. Nat Rev Clin Oncol 2017; 14: 169–86. doi: 10.1038/nrclinonc.2016.162 27725679 PMC5378302

[4] Cho N . Imaging features of breast cancer molecular subtypes: state of the art. J Pathol Transl Med 2021; 55: 16–25. doi: 10.4132/jptm.2020.09.03 33153242 PMC7829574

[5] Meyer-Bäse A, Morra L, Meyer-Bäse U, Pinker K . Current status and future perspectives of artificial intelligence in magnetic resonance breast imaging. Contrast Media Mol Imaging 2020; 2020: 6805710. doi: 10.1155/2020/6805710 32934610 PMC7474774

[6] Rahbar H, Partridge SC . Multiparametric MR imaging of breast cancer. Magn Reson Imaging Clin N Am 2016; 24: 223–38: S1064-9689(15)00110-5. doi: 10.1016/j.mric.2015.08.012 26613883 PMC4672390

[7] Tsarouchi MI, Vlachopoulos GF, Karahaliou AN, Vassiou KG, Costaridou LI . Multi-parametric MRI lesion heterogeneity biomarkers for breast cancer diagnosis. Phys Med 2020; 80: 101–10: S1120-1797(20)30249-0. doi: 10.1016/j.ejmp.2020.10.007 33137621

[8] Tan W, Yang M, Yang H, Zhou F, Shen W . Predicting the response to neoadjuvant therapy for early-stage breast cancer: tumor-, blood-, and imaging-related biomarkers. Cancer Manag Res 2018; 10: 4333–47. doi: 10.2147/CMAR.S174435 30349367 PMC6188192

[9] Galati F, Moffa G, Pediconi F . Breast imaging: beyond the detection. Eur J Radiol 2022; 146: 110051. doi: 10.1016/j.ejrad.2021.110051 34864426

[10] D’Orsi CJ, Sickles EA, Mendelson EB, Morris EA . ACR BI-RADS® Atlas, Breast Imaging Reporting and Data System. Reston, VA: American College of Radiology; 2013.

[11] Eisenhauer EA, Therasse P, Bogaerts J, Schwartz LH, Sargent D, Ford R, et al . New response evaluation criteria in solid tumours: revised RECIST guideline (version 1.1). Eur J Cancer 2009; 45: 228–47. doi: 10.1016/j.ejca.2008.10.026 19097774

[12] Fujiwara K, Yamada T, Kanemaki Y, Okamoto S, Kojima Y, Tsugawa K, et al . Grading system to categorize breast MRI in BI-RADS 5th edition: A multivariate study of breast mass descriptors in terms of probability of malignancy. American Journal of Roentgenology 2018; 210: W118–27. doi: 10.2214/AJR.17.17926 29381382

[13] Pfeiffer RM, Bur E . A model free approach to combining biomarkers. Biom J 2008; 50: 558–70. doi: 10.1002/bimj.200710428 18663762

[14] Moffa G, Galati F, Collalunga E, Rizzo V, Kripa E, D’Amati G, et al . Can MRI biomarkers predict triple-negative breast cancer? Diagnostics 2020; 10: 1090. 10.3390/diagnostics10121090 33333733 PMC7765199

[15] Öztürk VS, Polat YD, Soyder A, Tanyeri A, Karaman CZ, Taşkın F . The relationship between MRI findings and molecular subtypes in women with breast cancer. Current Problems in Diagnostic Radiology 2020; 49: 417–21. doi: 10.1067/j.cpradiol.2019.07.003 31351695

[16] Navarro Vilar L, Alandete Germán SP, Medina García R, Blanc García E, Camarasa Lillo N, Vilar Samper J . MR imaging findings in molecular subtypes of breast cancer according to BIRADS system. Breast J 2017; 23: 421–28. doi: 10.1111/tbj.12756 28067435

[17] Panzironi G, Moffa G, Galati F, Marzocca F, Rizzo V, Pediconi F . Peritumoral edema as a biomarker of the aggressiveness of breast cancer: results of a retrospective study on a 3 T scanner. Breast Cancer Res Treat 2020; 181: 53–60. doi: 10.1007/s10549-020-05592-8 32185587

[18] Demircioglu A, Grueneisen J, Ingenwerth M, Hoffmann O, Pinker-Domenig K, Morris E, et al . A rapid volume of interest-based approach of radiomics analysis of breast MRI for tumor decoding and phenotyping of breast cancer. PLoS ONE 2020; 15: e0234871. doi: 10.1371/journal.pone.0234871 32589681 PMC7319601

[19] Pinker K, Helbich TH, Morris EA . The potential of multiparametric MRI of the breast. BJR 2017; 90: 20160715. doi: 10.1259/bjr.20160715 27805423 PMC5605035

[20] Porembka JH, Ma J, Le‐Petross HT . Breast density, MR imaging biomarkers, and breast cancer risk. Breast J 2020; 26: 1535–42. doi: 10.1111/tbj.13965 32654416

[21] Niell BL, Abdalah M, Stringfield O, Raghunand N, Ataya D, Gillies R, et al . Quantitative measures of background parenchymal enhancement predict breast cancer risk. AJR Am J Roentgenol 2021; 217: 64–75. doi: 10.2214/AJR.20.23804 32876474 PMC9801515

[22] Baltzer PA, Dietzel M, Vag T, Burmeister H, Gajda M, Camara O, et al . Clinical MR mammography: impact of hormonal status on background enhancement and diagnostic accuracy. Rofo 2011; 183: 441–47. doi: 10.1055/s-0029-1246072 21318935

[23] Kinkel K . The never-ending success story of BI-RADS. Diagn Interv Imaging 2017; 98: 177–78. doi: 10.1016/j.diii.2017.02.003 28262125

[24] Bermot C, Saint-Martin C, Malhaire C, Sebbag-Sfez D, Mouret-Fourme E, Carton M, et al . Background parenchymal enhancement and fibroglandular tissue on breast MRI in women with high genetic risk: are changes before and after risk-reducing salpingo-oophorectomy associated with breast cancer risk? Eur J Radiol 2018; 109: 171–77. doi: 10.1016/j.ejrad.2018.10.030 30527300

[25] Liao GJ, Henze Bancroft LC, Strigel RM, Chitalia RD, Kontos D, Moy L, et al . Background parenchymal enhancement on breast MRI: A comprehensive review. J Magn Reson Imaging 2020; 51: 43–61. doi: 10.1002/jmri.26762 31004391 PMC7207072

[26] Dontchos BN, Rahbar H, Partridge SC, Korde LA, Lam DL, Scheel JR, et al . Are qualitative assessments of background parenchymal enhancement, amount of fibroglandular tissue on MR images, and mammographic density associated with breast cancer risk? Radiology 2015; 276: 371–80. doi: 10.1148/radiol.2015142304 25965809 PMC4554209

[27] Kim JY, Kim SH, Kim YJ, Kang BJ, An YY, Lee AW, et al . Enhancement parameters on dynamic contrast enhanced breast MRI: do they correlate with prognostic factors and subtypes of breast cancers? Magn Reson Imaging 2015; 33: 72–80. doi: 10.1016/j.mri.2014.08.034 25179138

[28] Hu N, Zhao J, Li Y, Fu Q, Zhao L, Chen H, et al . Breast cancer and background parenchymal enhancement at breast magnetic resonance imaging: a meta-analysis. BMC Med Imaging 2021; 21(1): 32. doi: 10.1186/s12880-021-00566-8 33607959 PMC7893738

[29] Uematsu T, Kasami M, Watanabe J . Does the degree of background enhancement in breast MRI affect the detection and staging of breast cancer? Eur Radiol 2011; 21: 2261–67. doi: 10.1007/s00330-011-2175-6 21688006

[30] DeMartini WB, Liu F, Peacock S, Eby PR, Gutierrez RL, Lehman CD . Background parenchymal enhancement on breast MRI: impact on diagnostic performance. AJR Am J Roentgenol 2012; 198: W373-80. doi: 10.2214/AJR.10.6272 22451576

[31] Bignotti B, Signori A, Valdora F . Evaluation of background parenchymal enhancement on breast MRI: a systematic review. Br J Radiol 2017; 90: 20160542. doi: 10.1259/bjr.20160542 27925480 PMC5685112

[32] Blaschke E, Abe H . MRI phenotype of breast cancer: kinetic assessment for molecular subtypes. J Magn Reson Imaging 2015; 42: 920–24. doi: 10.1002/jmri.24884 25758675

[33] O’Flynn EA, DeSouza NM . Functional magnetic resonance: biomarkers of response in breast cancer. breast cancer res. Erratum in: Breast Cancer Res 2011;13(3):405 2011. 10.1186/bcr2815 PMC3109577 21392409

[34] Yamaguchi K, Abe H, Newstead GM, Egashira R, Nakazono T, Imaizumi T, et al . Intratumoral heterogeneity of the distribution of kinetic parameters in breast cancer: comparison based on the molecular subtypes of invasive breast cancer. Breast Cancer 2014; 22: 496–502. doi: 10.1007/s12282-013-0512-0 24402638

[35] Ah-See M-LW, Makris A, Taylor NJ, Harrison M, Richman PI, Burcombe RJ, et al . Early changes in functional dynamic magnetic resonance imaging predict for pathologic response to neoadjuvant chemotherapy in primary breast cancer. Clin Cancer Res 2008; 14: 6580–89. doi: 10.1158/1078-0432.CCR-07-4310 18927299

[36] Pickles MD, Lowry M, Manton DJ, Gibbs P, Turnbull LW . Role of dynamic contrast enhanced MRI in monitoring early response of locally advanced breast cancer to neoadjuvant chemotherapy. Breast Cancer Res Treat 2005; 91: 1–10. doi: 10.1007/s10549-004-5819-2 15868426

[37] O’Connor JPB, Jackson A, Parker GJM, Jayson GC . DCE-MRI biomarkers in the clinical evaluation of antiangiogenic and vascular disrupting agents. Br J Cancer 2007; 96: 189–95. doi: 10.1038/sj.bjc.6603515 17211479 PMC2359994

[38] Iima M, Honda M, Sigmund EE, Ohno Kishimoto A, Kataoka M, Togashi K . Diffusion MRI of the breast: current status and future directions. J Magn Reson Imaging 2020; 52: 70–90. doi: 10.1002/jmri.26908 31520518

[39] Rizzo V, Moffa G, Kripa E, Caramanico C, Pediconi F, Galati F . Preoperative staging in breast cancer: intraindividual comparison of unenhanced MRI combined with digital breast tomosynthesis and dynamic contrast enhanced-MRI. Front Oncol 2021; 11: 661945. doi: 10.3389/fonc.2021.661945 34017683 PMC8130555

[40] Montemezzi S, Camera L, Giri MG, Pozzetto A, Caliò A, Meliadò G, et al . Is there a correlation between 3T multiparametric MRI and molecular subtypes of breast cancer? Eur J Radiol 2018; 108: 120–27. doi: 10.1016/j.ejrad.2018.09.024 30396643

[41] Moradi B, Gity M, Etesam F, Borhani A, Ahmadinejad N, Kazemi MA . Correlation of apparent diffusion coefficient values and peritumoral edema with pathologic biomarkers in patients with breast cancer. Clinical Imaging 2020; 68: 242–48. doi: 10.1016/j.clinimag.2020.08.020 32911312

[42] Guvenc I, Akay S, Ince S, Yildiz R, Kilbas Z, Oysul FG, et al . Apparent diffusion coefficient value in invasive ductal carcinoma at 3.0 tesla: is it correlated with prognostic factors? BJR 2016; 89: 20150614. doi: 10.1259/bjr.20150614 26853508 PMC4846196

[43] Bickel H, Pinker-Domenig K, Bogner W, Spick C, Bagó-Horváth Z, Weber M, et al . Quantitative apparent diffusion coefficient as a noninvasive imaging biomarker for the differentiation of invasive breast cancer and ductal carcinoma in situ. Investigative Radiology 2015; 50: 95–100. doi: 10.1097/RLI.0000000000000104 25333308

[44] Hamstra DA, Rehemtulla A, Ross BD . Diffusion magnetic resonance imaging: a biomarker for treatment response in oncology. JCO 2007; 25: 4104–9. doi: 10.1200/JCO.2007.11.9610 17827460

[45] Padhani AR, Liu G, Mu-Koh D, Chenevert TL, Thoeny HC, Takahara T, et al . Diffusion-weighted magnetic resonance imaging as a cancer biomarker: consensus and recommendations. Neoplasia 2009; 11: 102–25. doi: 10.1593/neo.81328 19186405 PMC2631136

[46] Pickles MD, Gibbs P, Lowry M, Turnbull LW . Diffusion changes precede size reduction in neoadjuvant treatment of breast cancer. Magn Reson Imaging 2006; 24: 843–47. doi: 10.1016/j.mri.2005.11.005 16916701

[47] Park SH, Moon WK, Cho N, Song IC, Chang JM, Park IA, et al . Diffusion-weighted MR imaging: pretreatment prediction of response to neoadjuvant chemotherapy in patients with breast cancer. Radiology 2010; 257: 56–63. doi: 10.1148/radiol.10092021 20851939

[48] Richard R, Thomassin I, Chapellier M, Scemama A, de Cremoux P, Varna M, et al . Diffusion-weighted MRI in pretreatment prediction of response to neoadjuvant chemotherapy in patients with breast cancer. Eur Radiol 2013; 23: 2420–31. doi: 10.1007/s00330-013-2850-x 23652844

[49] Woodhams R, Kakita S, Hata H, Iwabuchi K, Kuranami M, Gautam S, et al . Identification of residual breast carcinoma following neoadjuvant chemotherapy: diffusion-weighted imaging--comparison with contrast-enhanced MR imaging and pathologic findings. Radiology 2010; 254: 357–66. doi: 10.1148/radiol.2542090405 20093508

[50] Sharma U, Danishad KKA, Seenu V, Jagannathan NR . Longitudinal study of the assessment by MRI and diffusion-weighted imaging of tumor response in patients with locally advanced breast cancer undergoing neoadjuvant chemotherapy. NMR Biomed 2009; 22: 104–13. doi: 10.1002/nbm.1245 18384182

[51] Iacconi C, Giannelli M, Marini C, Cilotti A, Moretti M, Viacava P, et al . The role of mean diffusivity (MD) as a predictive index of the response to chemotherapy in locally advanced breast cancer: a preliminary study. Eur Radiol 2010; 20: 303–8. doi: 10.1007/s00330-009-1550-z 19760422

[52] Baltzer P, Mann RM, Iima M, Sigmund EE, Clauser P, Gilbert FJ, et al . Diffusion-weighted imaging of the breast-a consensus and mission statement from the EUSOBI international breast diffusion-weighted imaging working group. Eur Radiol 2020; 30: 1436–50. doi: 10.1007/s00330-019-06510-3 31786616 PMC7033067

[53] Yeung DK, Cheung HS, Tse GM . Human breast lesions: characterization with contrast-enhanced in vivo proton MR spectroscopy--initial results. Radiology 2001; 220: 40–46. doi: 10.1148/radiology.220.1.r01jl0240 11425970

[54] Fausto A, Magaldi A, Babaei Paskeh B, Menicagli L, Lupo EN, Sardanelli F . MR imaging and proton spectroscopy of the breast: how to select the images useful to convey the diagnostic message. Radiol Med 2007. 10.1007/s11547-007-0193-x 17952685

[55] Galati F, Luciani ML, Caramanico C, Moffa G, Catalano C, Pediconi F . Breast magnetic resonance spectroscopy at 3 T in biopsy-proven breast cancers: does choline peak correlate with prognostic factors? Invest Radiol 2019; 54: 767–73. doi: 10.1097/RLI.0000000000000597 31356383

[56] Gruber S, Debski B-K, Pinker K, Chmelik M, Grabner G, Helbich T, et al . Three-dimensional proton MR spectroscopic imaging at 3 T for the differentiation of benign and malignant breast lesions. Radiology 2011; 261: 752–61. doi: 10.1148/radiol.11102096 21998046

[57] Shin HJ, Baek H-M, Cha JH, Kim HH . Evaluation of breast cancer using proton MR spectroscopy: total choline peak integral and signal-to-noise ratio as prognostic indicators. AJR Am J Roentgenol 2012; 198: W488-97. doi: 10.2214/AJR.11.7292 22528931

[58] Pinker K, Bogner W, Baltzer P, Gruber S, Bickel H, Brueck B, et al . Improved diagnostic accuracy with multiparametric magnetic resonance imaging of the breast using dynamic contrast-enhanced magnetic resonance imaging, diffusion-weighted imaging, and 3-dimensional proton magnetic resonance spectroscopic imaging. Invest Radiol 2014; 49: 421–30. doi: 10.1097/RLI.0000000000000029 24566292

[59] Begley JKP, Redpath TW, Bolan PJ, Gilbert FJ . In vivo proton magnetic resonance spectroscopy of breast cancer: a review of the literature. Breast Cancer Res 2012; 14: 207. doi: 10.1186/bcr3132 22515594 PMC3446370

[60] Jagannathan NR, Kumar M, Seenu V, Coshic O, Dwivedi SN, Julka PK, et al . Evaluation of total choline from in-vivo volume localized proton MR spectroscopy and its response to neoadjuvant chemotherapy in locally advanced breast cancer. Br J Cancer 2001; 84: 1016–22. doi: 10.1054/bjoc.2000.1711 11308247 PMC2363867

[61] Sharma U, Baek HM, Su MY, Jagannathan NR . In vivo 1H MRS in the assessment of the therapeutic response of breast cancer patients. NMR Biomed 2011; 24: 700–711. doi: 10.1002/nbm.1654 21793075 PMC4226268

[62] Danishad KKA, Sharma U, Sah RG, Seenu V, Parshad R, Jagannathan NR, et al . Assessment of therapeutic response of locally advanced breast cancer (LABC) patients undergoing neoadjuvant chemotherapy (NACT) monitored using sequential magnetic resonance spectroscopic imaging (MRSI). NMR Biomed 2010; 23: 233–41. doi: 10.1002/nbm.1436 20175134

[63] Thakur SB, Horvat JV, Hancu I, Sutton OM, Bernard-Davila B, Weber M, et al . Quantitative in vivo proton MR spectroscopic assessment of lipid metabolism: value for breast cancer diagnosis and prognosis. J Magn Reson Imaging 2019; 50: 239–49. doi: 10.1002/jmri.26622 30605266 PMC6579700

[64] Ramadan S, Arm J, Silcock J, Santamaria G, Buck J, Roy M, et al . Lipid and metabolite deregulation in the breast tissue of women carrying BRCA1 and BRCA2 genetic mutations. Radiology 2015; 275: 675–82. doi: 10.1148/radiol.15140967 25734415

[65] Barzilai A, Horowitz A, Geier A, Degani H . Phosphate metabolites and steroid hormone receptors of benign and malignant breast tumors. A nuclear magnetic resonance study. Cancer 1991; 67: 2919–25. doi: 10.1002/1097-0142(19910601)67:11<2919::aid-cncr2820671135>3.0.co;2-z 1851051

[66] Codari M, Schiaffino S, Sardanelli F, Trimboli RM . Artificial intelligence for breast MRI in 2008-2018: A systematic mapping review. AJR Am J Roentgenol 2019; 212: 280–92. doi: 10.2214/AJR.18.20389 30601029

[67] Gillies RJ, Kinahan PE, Hricak H . Radiomics: images are more than pictures, they are data. Radiology 2016; 278: 563–77. doi: 10.1148/radiol.2015151169 26579733 PMC4734157

[68] Galati F, Trimboli RM, Pediconi F . Special issue “advances in breast mri.” Diagnostics (Basel) 2021; 11(12): 2297. 10.3390/diagnostics11122297 34943534 PMC8700161

[69] Rogers W, Thulasi Seetha S, Refaee TAG, Lieverse RIY, Granzier RWY, Ibrahim A, et al . Radiomics: from qualitative to quantitative imaging. Br J Radiol 2020; 93(1108): 20190948. doi: 10.1259/bjr.20190948 32101448 PMC7362913

[70] Kumar V, Gu Y, Basu S, Berglund A, Eschrich SA, Schabath MB, et al . Radiomics: the process and the challenges. Magn Reson Imaging 2012; 30: 1234–48. doi: 10.1016/j.mri.2012.06.010 22898692 PMC3563280

[71] Valdora F, Houssami N, Rossi F, Calabrese M, Tagliafico AS . Rapid review: radiomics and breast cancer. Breast Cancer Res Treat 2018; 169: 217–29. doi: 10.1007/s10549-018-4675-4 29396665

[72] Grimm LJ . Breast MRI radiogenomics: current status and research implications. J Magn Reson Imaging 2016; 43: 1269–78. doi: 10.1002/jmri.25116 26663695

[73] Pinker K, Shitano F, Sala E, Do RK, Young RJ, Wibmer AG, et al . Background, current role, and potential applications of radiogenomics. J Magn Reson Imaging 2018; 47: 604–20. doi: 10.1002/jmri.25870 29095543 PMC5916793

[74] Zhu Y, Li H, Guo W, Drukker K, Lan L, Giger ML, et al . Deciphering genomic underpinnings of quantitative MRI-based radiomic phenotypes of invasive breast carcinoma. Sci Rep 2015; 5: 17787. doi: 10.1038/srep17787 26639025 PMC4671006

[75] Bismeijer T, van der Velden BHM, Canisius S, Lips EH, Loo CE, Viergever MA, et al . Radiogenomic analysis of breast cancer by linking MRI phenotypes with tumor gene expression. Radiology 2020; 296: 277–87. doi: 10.1148/radiol.2020191453 32452738

[76] Woodard GA, Ray KM, Joe BN, Price ER . Qualitative radiogenomics: association between oncotype DX test recurrence score and BI-RADS mammographic and breast MR imaging features. Radiology 2018; 286: 60–70. doi: 10.1148/radiol.2017162333 28885890

[77] Yin X-X, Hadjiloucas S, Zhang Y, Tian Z . MRI radiogenomics for intelligent diagnosis of breast tumors and accurate prediction of neoadjuvant chemotherapy responses-a review. Comput Methods Programs Biomed 2022; 214: 106510. doi: 10.1016/j.cmpb.2021.106510 34852935

[78] Jakhar D, Kaur I . Artificial intelligence, machine learning and deep learning: definitions and differences. Clin Exp Dermatol 2020; 45: 131–32. doi: 10.1111/ced.14029 31233628

[79] Reig B, Heacock L, Geras KJ, Moy L . Machine learning in breast MRI. J Magn Reson Imaging 2020; 52: 998–1018. doi: 10.1002/jmri.26852 31276247 PMC7085409

[80] Tahmassebi A, Wengert GJ, Helbich TH, Bago-Horvath Z, Alaei S, Bartsch R, et al . Impact of machine learning with multiparametric magnetic resonance imaging of the breast for early prediction of response to neoadjuvant chemotherapy and survival outcomes in breast cancer patients. Invest Radiol 2019; 54: 110–17. doi: 10.1097/RLI.0000000000000518 30358693 PMC6310100

[81] Hu Q, Whitney HM, Giger ML . A deep learning methodology for improved breast cancer diagnosis using multiparametric MRI. Sci Rep 2020; 10(1): 10536. doi: 10.1038/s41598-020-67441-4 32601367 PMC7324398

[82] Sheth D, Giger ML . Artificial intelligence in the interpretation of breast cancer on MRI. J Magn Reson Imaging 2020; 51: 1310–24. doi: 10.1002/jmri.26878 31343790

